# The Role of the DNA Damage Response throughout the Papillomavirus Life Cycle

**DOI:** 10.3390/v7052450

**Published:** 2015-05-21

**Authors:** Caleb C. McKinney, Katherine L. Hussmann, Alison A. McBride

**Affiliations:** Laboratory of Viral Diseases, National Institute of Allergy and Infectious Diseases, National Institutes of Health, Bethesda, MD 20892, USA; E-Mails: caleb.mckinney@nih.gov (C.C.M.); katherine.hussmann@nih.gov (K.L.H.)

**Keywords:** HPV, papillomavirus, replication, DNA damage response, homologous recombination, keratinocyte, replication foci, DNA repair, partitioning, episome

## Abstract

The DNA damage response (DDR) maintains genomic integrity through an elaborate network of signaling pathways that sense DNA damage and recruit effector factors to repair damaged DNA. DDR signaling pathways are usurped and manipulated by the replication programs of many viruses. Here, we review the papillomavirus (PV) life cycle, highlighting current knowledge of how PVs recruit and engage the DDR to facilitate productive infection.

## 1. Introduction

Papillomaviruses (PVs) are an ancient group of small, double-stranded (ds) DNA viruses that infect the highly adapted niche of the stratified epithelium of the skin or mucosa in specific host species. Although HPV infection can be asymptomatic, HPVs are also the etiological agent of a wide range of benign papillomas or warts [1]. A subset of HPVs cause infections that can transition to cancer after long-term, persistent infection [2,3]. As the causative agent of over 99% of cervical cancers and an increasing number of anogenital and oropharyngeal cancers, HPVs remain a serious health threat [4].

**Figure 1:**
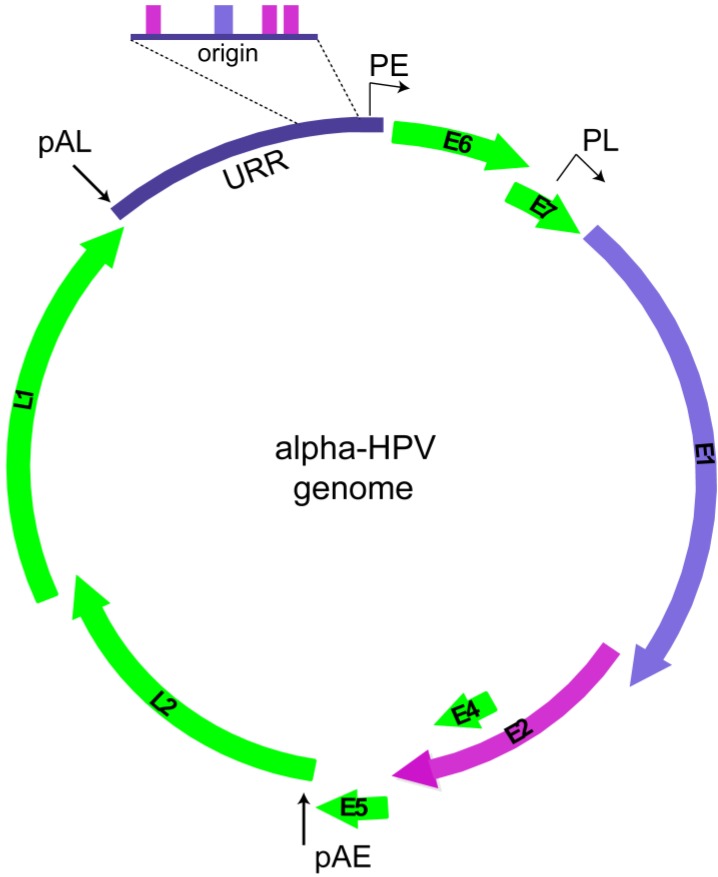
Viral genome. The circular dsDNA genome of an alpha-HPV genome is shown. Viral open reading frames are depicted as curved arrows. The URR (upstream regulatory region) is expanded to show the replication origin containing binding sites for the E2 protein (magenta boxes) and the E1 binding site (purple rectangle) are shown. The early promoter (PE), late promoter (PL) and early and late polyadenylation sites (pAE and pAL) are indicated.

There are several hundred different PVs that infect a multitude of individual host species, yet the basic genomic scheme and life cycle strategy of each virus are remarkably consistent. Each virus contains a small dsDNA circular genome of approximately 8 kb that encodes just six to eight genes [5] (Figure 1).

Because of this limited coding capacity, PVs retain a strict dependence on host factors for viral replication [6]. Elucidation of the different replication mechanisms of HPV could identify novel targets that may prove effective in the development of therapies for preexisting HPV infections, where the multivalent vaccines are not readily useful. Recent studies have shown that the DNA damage response and repair (DDR) machinery is necessary for efficient HPV replication. Here, we review the mechanisms of genome replication in the HPV life cycle and summarize how the virus recruits and engages the DDR to facilitate viral DNA synthesis.

## 2. Replication Phases in the Papillomavirus Life Cycle

### 2.1. Overview of Phases of Replication in Papillomavirus Infection

**Figure 2:**
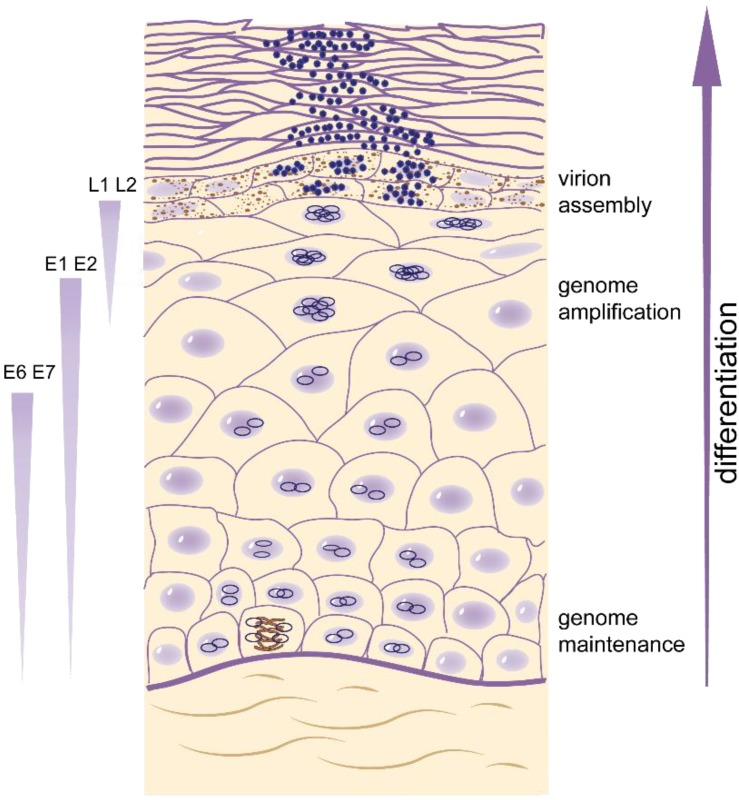
Model of a stratified epithelium. The layers of epithelium shown from bottom to top are stratum basale (with a mitotic cell), stratum spinosum, stratum granulosum (with brown keratohyalin granules), and stratum corneum. The dividing cells in the lowest layer of the epithelium maintain the viral genome as a plasmid (dark blue circles). As these cells progress upwards during the process of differentiation, viral genomes are amplified and packaged in viral particles (dark blue particles). The levels of viral proteins also increase with differentiation as shown on the left.

Upon initial infection of cells in the basal epithelium, the HPV genome enters the nucleus and undergoes a limited number of rounds of DNA replication to establish a low copy number of genomes per infected cell. Subsequently, when the infected cells in the basal layer replicate and divide, the HPV genome replicates in synchrony as extrachromosomal elements tethered to the host genome. When these infected cells detach from the basement membrane and enter the suprabasal epithelial layer as part of the differentiation process, the HPV E6 and E7 proteins promote unscheduled cell cycle progression by manipulating cell cycle regulation and cellular differentiation programs (see Figure 2). As cells continue to advance through the epithelial layer, there is a switch in the mode of genome replication to productive viral DNA amplification concomitant with increased levels of the E1 and E2 replication proteins. In the terminally differentiated layers of the epithelium, L1 and L2, the viral capsid proteins, are synthesized, and viral particles are assembled.

### 2.2. HPV Replication Proteins

The relatively small intrinsic coding capacity of HPV genomes warrants exquisite dependence on host factors, as well as viral factors, for replication. HPV encodes two proteins directly involved in replication: E1, a replicative helicase, and E2, a multifunctional protein that recruits E1 to the replication origin of the viral genome [7,8,9]. The replication origin, located in the Upstream Regulatory Region (URR) of the viral genome, contains binding sites for both E1 and E2 proteins [9]. In cooperation with E2, E1 binds specifically to the replication origin of HPV, but E2 is then displaced and E1 converts to a double hexamer that unwinds the DNA in an ATP-dependent manner [10]. The host DNA replication machinery is then recruited to synthesize the viral DNA. Both E1 and E2 are required for initial amplification and establishment of viral DNA and late vegetative replication [11]. However, in certain circumstances, E1 is dispensable for maintenance replication [11,12].

Additional HPV proteins are required to maintain an environment conducive to viral replication. The oncogenic HPV E6 and E7 proteins promote cellular proliferation, delay cellular differentiation, and promote immune evasion (reviewed in [2]). Both E6 and E7 are required for productive replication [13,14,15] and it was long thought that E6 and E7 were required to sustain differentiated cells in a pseudo-S phase to provide access to host DNA replication machinery. However, in a stratified epithelium, the differentiated cells that amplify viral genomes are in a G2 like phase that is also dependent on E7 expression [16,17]. As described below, E6 and E7 play essential roles in modulating the cellular DDR for viral replication.

### 2.3. Initial Amplification and Establishment

HPVs infect the dividing keratinocytes of the basal layer by entering the stratified epithelium through a microabrasion (reviewed in [18]). Virions initially bind through interactions with heparin sulfate proteoglycans [19], and the viral capsid is processed and trafficked through the endosomal pathway. The minor capsid protein, L2, is bound to the viral genome within the virion and, once the virion is processed through the endosome, delivers the genome into the nucleus where it localizes to ND10 bodies [20]. Nuclear access most likely occurs in mitotic cells, where nuclear envelope breakdown allows nuclear access, rather than entry through a nuclear pore [21,22].

**Figure 3:**
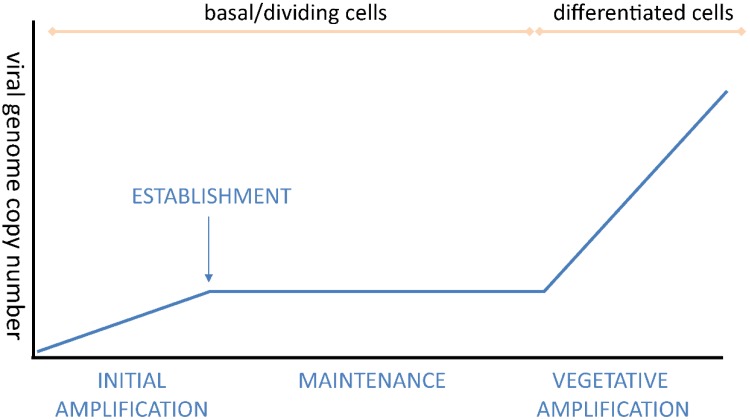
Viral Genome Copy Number during the Different Phases of Replication.

Once in the nucleus, the viral genome replicates to a low level number of copies per cell. The genome copy number in the basal cells is low, as the viral genome cannot be detected by *in situ* techniques during this stage [23,24]. The infected cell maintains this low copy number as the viral DNA replicates during subsequent cell divisions in the maintenance replication phase (Figure 3).

A time course of HPV31 infection in HaCaT cells demonstrated that spliced messages which could encode E1 and E2 were present just four hours post-infection [25], consistent with the requirement of E1 and E2 for initial replication. Other viral transcripts appear at eight hours post-infection [25]. The E8^E2 transcript was also detected at four hours post-infection and this is very consistent with its role as a repressor of viral transcription and replication [26]. Expression of E8^E2 at this stage of infection could temper runaway replication and promote the switch to maintenance replication.

### 2.4. Maintenance Replication and Persistence

During the maintenance phase of replication, the viral genome is replicated in S-phase along with the host genome. Initial evidence using bovine papillomavirus type 1 (BPV1) suggested that viral genome replication is licensed during the maintenance phase [27,28], but later studies demonstrated that in S-phase cells, replication is by a random choice mechanism, whereby some genomes undergo multiple rounds of replication and others remain unreplicated [29,30]. A more recent study of cell lines maintaining HPV genomes revealed that, depending on the HPV type and cell line, both replication mechanisms could be detected in dividing cells [31]. High levels of E1 expression promoted random choice replication, which likely represents the unscheduled DNA synthesis characteristic of vegetative amplification [31].

The level of replication must be tightly controlled during the maintenance phase of the viral life cycle and this may occur via several mechanisms. The E1 protein is retained in the cytoplasm of cells that are not undergoing S-phase [32,33] and in certain circumstances the E1 protein might even be dispensable for maintenance replication [11,12]. The E8^E2 transcriptional repressor tightly regulates both transcription and replication and is required to regulate maintenance replication [26,34]. HPVs also regulate microRNA 145, which in turn binds to the E1 and E2 genes and down-regulates their expression [35]. In BPV1, E2 is a limiting factor in maintenance replication and phosphorylation of the E2 protein regulates its stability and modulates genome copy number in dividing cells [36].

For HPV to persist in dividing cells, not only must the viral genomes be replicated in synchrony with host DNA, but nascent genomes must be efficiently partitioned to daughter cells. The partitioning model, best defined for BPV1, is that the E2 protein binds to multiple E2 binding motifs within the viral URR and tethers the viral genome to host chromatin, thus ensuring it is perpetuated within the basal layer of cells [37,38,39]. While this model likely applies to all PVs, it is probable that there are differences in the details [40]. For example, most HPVs do not have the large number of E2 binding sites found in BPV1 (and other delta PVs) and all PV E2 proteins do not bind tightly to host mitotic chromatin [41]. Nevertheless, the tethering strategy is likely to be universal for all PVs, as a similar mechanism is used by other persistent viruses that maintain their genomes as extrachromosomal plasmids.

Attachment of PV genomes to host chromatin is important for more than just partitioning of genomes to daughter cells because tethering to active, inactive or even genetically unstable regions of host chromatin could have important outcomes for the infection [42,43,44]. Extensive studies of the E2 protein have shown its interaction with several well characterized proteins involved in replication, transcription, and host cell cycle regulation [45] and reviewed in [46], and several proteins have been proposed to be the chromatin target responsible for partitioning the E2-PVgenome complex (reviewed in [46]). One of the best-studied (and most debated) targets is BRD4, a chromatin adaptor protein essential for transcriptional initiation and elongation, as well as mitotic bookmarking of host chromatin (reviewed in [47,48]). E2 binds to BRD4 and stabilizes its association with host chromatin [49,50,51], and BRD4 is a key regulator of PV transcription [52,53,54,55,56]. However, the role of BRD4 in the partitioning of the alpha-PV genomes seems to be more complex since these E2 proteins bind with lower affinity to BRD4 (and to host chromatin) [45,52]. Alpha-E2s and BRD4 are not observed on host mitotic chromosomes under conditions in which other E2 proteins are readily detected; however, the HPV16 E2-BRD4 complex has recently been discovered on chromosomes by bimolecular fluorescence complementation [57] or when E2 is expressed as a GFP-fusion protein [58]. However, HPV31 genomes that encode an E2 protein defective for BRD4 binding are maintained in stable cell lines [59,60], suggesting either that BRD4 is not essential for genome maintenance of all HPV strains, or that the interaction of BRD4 and E2 is more complex in the background of a viral infection. Accordingly, there is evidence that the latter situation is true, since BRD4 is associated with replication foci formed both by E1 and E2 overexpression and in HPV31 genome-containing differentiated cells [61,62,63]. In the latter situation BRD4 surrounds the replication foci in a satellite pattern [61], but its exact mechanistic role is not clear. BRD4 could facilitate viral transcription in replication factories, could directly function in replication, or might interface with components of the DDR. There are hints that BRD4 might function in the DDR; it interacts with the DDR associated RFC1 subunit, ATAD5 [53], a minor isoform of BRD4 protects adjacent chromatin from DNA damage signaling [64], and we find that BRD4 is associated with aphidicolin inducible fragile sites in C-33A cells [43,64]. Thus, BRD4, and possibly other host proteins required for maintenance replication, may play complex roles during the transition from maintenance replication to vegetative amplification.

### 2.5. Differentiation-Dependent Viral DNA Amplification

The third phase of HPV replication is tightly linked to the differentiation program of the stratified epithelium. HPVs must transition out of maintenance phase to induce high level viral DNA synthesis, late gene expression, and production and perpetuation of infectious particles. As proliferating cells that harbor HPV genomes transition through the differentiation process, the late promoter is activated, high levels of the E1 and E2 proteins are expressed and the mode of viral genome replication switches to support productive viral genome amplification (Figure 2 and Figure 3 and [65]). Using laser microdissection to analyze viral genome copy number of OcPV1 (ROPV; rabbit oral papillomavirus) in the stratified layers of oral lesions, Maglennon *et al.* demonstrate a five log amplification of viral DNA in the superficial layers compared to the basal layers [66].

The exact mechanism of genome amplification has not been elucidated, but there has long been evidence that it might differ from the bidirectional theta mode characteristic of maintenance replication [67,68]. A breakthrough in the study of HPV replication was the observation that differentiation-dependent genome amplification required the cellular ATM DNA damage response and repair (DDR) pathway [69]. This finding opened up the possibility that genomes might be amplified in differentiated cells by a recombination-dependent replication mode supported by the DDR response.

The remainder of this review will focus on recent developments that uncover how HPV contests and hijacks the carefully crafted DDR network to recruit, engage, and manipulate the host DDR machinery for viral DNA replication at various stages of the life cycle.

## 3. Role of the DNA Damage Response (DDR) in HPV Replication

### 3.1. DNA Damage Response Overview

**Figure 4:**
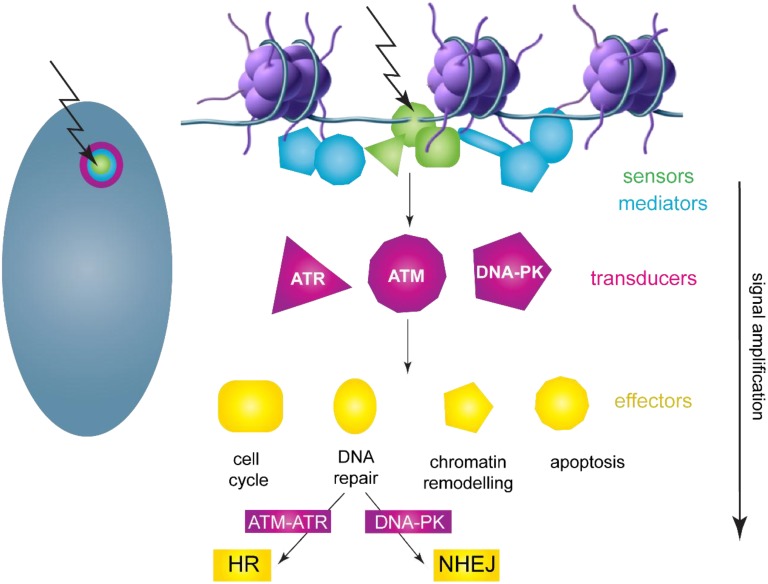
Diagram of the DNA Damage and Repair Response Pathway. DNA breaks or collapsed replication forks are detected by sensors that mark the site of damage. Three kinase signaling cascades (ATM, ATR, and DNA-PK) regulate and transduce the response to damage. With the help of mediator proteins, transducer and effector proteins transmit and greatly amplify the signal throughout the cell, resulting in a huge influx of factors to repair damage and remodel chromatin. Two major repair pathways, HR and NHEJ, are regulated by the ATM/ATR and DNA-PK pathways, respectively. The nucleus on the left contains an HPV replication focus that mimics a DDR focus. Adapted from [71].

Cellular DNA constantly sustains endogenous and exogenous insults that result in mutations, crosslinks and single and double stranded breaks [70]. However, cells have evolved an elaborate pathway to correct these lesions known as the DNA damage and repair response (DDR) (Figure 4 and reviewed in [71]). The DDR consists of sensors, such as the MRE11-Rad50-Nbs1 complex (MRN) or replication protein A (RPA), that detect a wide range of DNA lesions and recruit the Ataxia telangiectasia mutated (ATM) and Ataxia telangiectasia and Rad3 related (ATR) transducer kinases to the site of damage. With the help of mediator proteins, these transducer kinases induce a signaling cascade through the effector kinases Chk1 and Chk2, activating cell cycle checkpoints to prevent cell cycle progression and ultimately recruiting the DNA repair machinery to the damaged DNA. Once the damage is repaired, the checkpoints are mitigated and cells return to their normal state. However, extensive DNA damage may warrant the initiation of cellular death pathways (Figure 4).

Some of the most severe types of damage are dsDNA breaks that result from ionizing radiation, replication stress, reactive metabolic intermediates, or exogenous chemicals and result in activation of the ATM pathway. dsDNA breaks are bound by the MRN complex that both bridges the gap and activates the ATM signaling cascade. This results in activation of a Chk2 dependent cell cycle checkpoint. dsDNA breaks can be repaired either through the low fidelity, non-homologous end joining (NHEJ) pathway or the high fidelity, homologous recombination (HR) pathway. In HR, a section of DNA adjacent to the 5′ end of the break is resectioned by an exonuclease complex recruited by the MRN complex. This ssDNA becomes coated with Rad51 and invades the undamaged sister chromatid, which is used to provide a template for DNA synthesis to faithfully restore the sequence on the damaged strand. Since a homologous DNA sequence is required for proper repair, HR is restricted to S and G2 phases of the cell cycle, when a homologous sister chromatid is present to provide a template.

ATR is activated in response to the presence of persistent ssDNA that results from replication stress. RPA (replication protein A) binds this ssDNA and recruits ATRIP (ATR interacting partner) and ATR. The ring-shaped 9-1-1 complex (consisting of Rad9, Hus1, Rad1) is loaded onto collapsed replication forks and recruits TopBP1 (Topoisomerase II-binding protein 1), a multifaceted factor essential for maintaining genomic stability and facilitating DNA replication by recruiting replication factors to replication forks. 9-1-1 further enlists claspin, which recruits the kinase, Chk1. ATR-dependent phosphorylation of Chk1 activates a cell cycle checkpoint and facilitates stabilization of replication forks. The DDR pathway not only functions to accurately repair DNA, but must also regulate the cell cycle, pausing it to allow repair to be completed. The DDR activates specific downstream cell cycle checkpoints during each phase of the cell cycle to maintain DNA integrity. For example, a DDR mediated increase in stability of p53 can lead to increased expression of CDK inhibitor p21, which arrests cells in G1. Likewise, Chk1 and Chk2 phosphorylation inhibits Cdc25 family members, which are important for progression at several stages of the cell cycle (reviewed in [72]).

The unusual structure of viral DNA (e.g., extrachromosomal linear or small circular molecules), unscheduled viral DNA synthesis, viral protein expression or aberrant cellular proliferation can induce a DDR in infected cells. While this can be detrimental to infection, many viruses disable certain components of the DDR and take advantage of others to synthesize and process viral DNA in productive infection [73,74]. Viral DNA synthesis can also result in additional unique structures that require processing. For example, studies in SV40 show that ATM and ATR are required for quality control of viral replication products and for maintenance of the replication foci; ATM signaling reduces unidirectional replication and ATR signaling prevents DNA breakage at the converging forks that result from bidirectional replication [75]. ATM also ensures that HR factors, and not NHEJ factors, are recruited to SV40 replication centers [76]. The use of repair pathways to synthesize viral DNA also enables replication in cells that are outwith S-phase. Not only does this reduce competition from host cell synthesis, but in the case of HPVs, the use of repair pathways to synthesize DNA allows replication in differentiated cells. A landmark study in the HPV field demonstrated that the ATM arm of the DDR response was required for amplification of HPV31 genomes in differentiated cells [69]. Subsequent studies have shown that several viral proteins, including E1 and E7, are involved in the recruitment of DSB repair factors to HPV replication factories [61,77,78,79,80]. HPV replication foci also recruit factors from both the ATM and ATR pathways, such as TopBP1, Rad51, pNBS1, MRN, RPA, BRCA1, and 53BP1 [61,63,69,77,78,79,80,81].

In the context of the life cycle, E6 and E7 play important roles in both promoting DDR pathways as well as inhibiting the downstream consequences. In general, E7 promotes the DDR to provide an environment conducive for viral replication while E6 mitigates the downstream effects of the DDR cascade to allow cells to tolerate chronic DDR activation, growth arrest and/or apoptosis. Thus, HPVs have usurped DNA damage sensing and repair strategies to promote viral DNA replication. The sections below will review current knowledge of the role of the DDR in the different phases of HPV DNA replication.

### 3.2. HPV Replication Foci Formation

High level amplification of viral DNA occurs as infected cells differentiate and progress toward the epithelial surface. Most viruses replicate their genomes in defined cellular regions that are designated replication foci, compartments, or factories. These regions serve to nucleate and concentrate the components required for viral DNA synthesis and other processes to specific regions of the cell, presumably to facilitate viral replication. Nuclear replication foci can be detected in differentiated cells that contain replicating HPV [61,69,82].

Studies from our lab as well as others have shown that HPV viral DNA replication occurs in nuclear foci and is dependent on the E1 and E2 proteins. Expression of the E1 and E2 replication proteins (along with an origin containing replicon) is sufficient for the formation of replication foci that recruit components of the DDR [77,78,80,83]. The E1 protein induces the DDR and this is concentrated into nuclear foci by the E2 protein [77,78]. Viral DNA is amplified to high levels in these foci and this does not require keratinocyte differentiation, most likely because E1 and E2 are provided from heterologous promoters. Remarkably, viral replication factories mimic cellular DNA damage foci, whereby a single DNA lesion results in a huge influx of factors to repair damage (Figure 4). Thus, by inducing and usurping the DDR pathways, HPVs can take advantage of major cellular pathways and resources

### 3.3. The Role of the DNA Damage Response during Initial Amplification of HPV

Difficulties in obtaining large numbers of virions have greatly hampered the study of the early events of HPV infection. Viral DNA (cleaved from plasmids propagated in bacteria and recircularized) can be transfected into keratinocytes or other cells as a surrogate for early infection. Such studies may not completely mimic delivery of a viral genome in a virion particle, but they can provide some insight into the early events required for infection. On the other hand, replication obtained with viral genomes deficient in E8^E2 repressor expression, or when viral genomes are co-transfected with E1 and E2 expression vectors, most likely represent the runaway replication observed in vegetative amplification.

It has been shown that PVs enter the nucleus and initiate their replication and transcription program adjacent to ND10 bodies, like many other viruses [20]. ND10 bodies are important for anti-viral defense and one of the components, Sp100, represses transcription and replication of incoming virions [84]. ND10 bodies are associated with regions of DNA damage in uninfected cells and may provide a link between early events in HPV entry and the initial rounds of unscheduled viral DNA synthesis required to initiate infection [85]. As HPV enters the cell it is likely that this unscheduled viral DNA synthesis will activate or use components of the DDR for limited amplification of the viral genome. A recent study from our laboratory demonstrated that the HPV E2 proteins bind to host chromatin in complex with BRD4 at regions of the genome undergoing replication stress [43], and we proposed that these interactions are important for both genome tethering during maintenance replication as well as nucleation of replication foci in vegetative amplification. We predict that the interaction of viral genomes with host chromatin becomes established at very early stages of infection and involves the DNA damage response.

Although it has been shown in some studies that transient replication of HPV16 is not inhibited by DNA damage signaling or by p53 induction [86,87], it is likely that these responses have to be tempered for the infected cell to transition into the maintenance phase. A quantitative colony-forming assay is a useful measure of genome establishment [84,88], but it indicates that this is a rare event. This is very similar to findings that establishment of Epstein-Barr virus derived replicons is infrequent [89]. Thus, the virus must evade intrinsic immune responses, modulate DDR responses, and tether to beneficial regions of host chromatin to ensure a long-term, persistent infection.

### 3.4. The Role of the DNA Damage Response during Maintenance Replication.

During the maintenance phase of replication, HPV genomes are replicated in S-phase in synchrony with host DNA replication. Cells containing HPV genomes have increased markers of ATM and ATR signaling, but pharmacological inhibition of the ATM pathway does not affect genome maintenance [69]. However, it has been reported that siRNA-mediated reduction of ATM, ATR or several other proteins in the DDR pathway, as well as Chk1 inhibition results in 40%–50% reduction in HPV16 copy number in W12 cells [90,91].

Many DNA tumor viruses inactivate pRB and p21 and derepress expression of the E2F transcription factor family and for HMCV this is important for ATM-driven viral replication [92]. Furthermore, deregulation of E2F1 by E7 induces the expression of Chk2 [93], implying that this function of E7 could promote HPV replication. E7 deregulation of E2F, and subsequent nucleotide deficiency, further promotes replication stress and genomic instability [94]. In addition, E7 can promote mitotic entry in the presence of a DDR response by accelerating the degradation of the Chk1 binding protein, claspin [95]. Therefore, HPVs integrate DNA damage signaling with anti-apoptotic and proliferative functions to create a unique cellular environment that supports viral replication.

Activation of ATM and ATR signaling results in activation of p53 with a concomitant growth arrest that would be detrimental for viral replication in dividing cells. However, several studies have shown that the ability of E6 to degrade and/or inactivate p53 is crucial for long-term genome maintenance [14,96,97]. Furthermore, inactivation of p53 or expression of a dominant negative p53 protein can complement genomes defective for E6 expression [87,98].

### 3.5. The Role of the DNA Damage Response during Vegetative Amplification of Viral Genomes

Most of what we know about the role of the DDR in HPV biology has been elucidated from studies of late viral DNA replication. The E1 and E2 replication proteins are induced to high levels by a differentiation-dependent induction of the late promoter [99] and this coincides with activation of a DDR [69]. Many studies have also shown that E1 can specifically upregulate and contribute to the activation of the DDR in non-differentiated cells, and when coexpressed with E2, many DDR factors such as pATM, pATR, γH2AX, pChk2, pChk1, BRCA1, RAD51, TopBP1 and pNBS1 colocalize within nuclear foci that replicate viral DNA to high levels [63,77,78,80].

In the more natural condition of a stratified epithelium, E7 plays an important role in inducing the ATM arm of the DDR in differentiated cells [16] and ATM signaling is required for genome amplification [69]. E7 specifically interacts with NBS1 (which is also required for viral replication), and this association can be separated from activation of the ATM pathway [100]. Further, studies using inhibitors of signal transducer and transactivator 5 (STAT5), a member of the JAK-STAT pathway and an important regulator of the immune response, displayed that STAT5 positively regulates vegetative amplification of HPV31 via potentiation of the ATM pathway through activation of Chk2 [101]. STAT5 activity was dependent on E7 expression, further demonstrating the role of E7 during vegetative amplification. Although the extent of the landscape of E7-driven regulation of cellular protein expression and signaling pathways is not fully understood, E7-mediated upregulation and accumulation of MRN and HR factors as well as stimulation of ATM through the activity of STAT5 is required to properly engage the DDR machinery to facilitate differentiation-dependent genome amplification, bridging the nexus of innate immunity and DNA repair.

The dysregulation of p53 via E6 during vegetative amplification is also necessary to provide an environment conducive for viral DNA amplification. Viruses that are unable to degrade or inactivate p53 are unable to amplify viral DNA in a stratified epithelium [102]. Separate from its role in checkpoint control, p53 can directly inhibit amplification replication in several PVs [103,104,105], perhaps by directly binding to the E2 protein [106]. Therefore, like other viruses, HPVs take advantage of some aspects of the DDR, but inactivate others.

The presence of several HR factors within HPV replication foci indicates that the transition from maintenance to amplification might also involve a switch to a distinct replication mechanism, such as recombination-dependent replication [67,68,82,107,108]. This has several advantages such as enabling replication in the G2 phase of the cell cycle, as well as generating large amounts of viral DNA without the need for replication initiator proteins to reinitiate DNA synthesis [109].

### 3.6. The Role of the DNA Damage Response in HPV Integration and Carcinogenesis

As described above, E7 and E6 have important roles in promoting the DDR to support viral DNA replication and render cells resistant to the consequences of checkpoint activation. However, this inadvertently leaves cells highly susceptible to mutation and genetic instability. This has been well studied for the oncogenic alpha-HPVs, but there is also evidence that the cutaneous beta-HPVs might manipulate the host DDR pathways to promote viral replication. Indeed, expression of beta-HPV E6 interferes with DDR signaling at many levels, rendering cellular genomes vulnerable to UV damage and carcinogenic genetic instability [110,111,112,113,114,115,116].

In many HPV associated cancers, the viral genome has become integrated into the host genome [117] often in regions of genetic instability called common fragile sites [118,119]. Could this be related to the reliance of the virus on the DDR for replication? We have shown recently that HPV E2 proteins associate with genetically unstable regions of the host chromosomes and also that late replication foci form adjacent to these sites [43]. It could be beneficial to the virus to associate with regions of host chromatin that are highly susceptible to replication stress and prone to late replication. However, the close association of viral and host replication (especially regions undergoing recombination-directed repair) could inadvertently lead to integration of the viral genome. Notably, common fragile sites and extrachromosomal HPV genomes have similar susceptibilities to ATR inhibitors, and to the replication inhibitor aphidicolin [90]. Recent studies have shown that HPV integration sites are highly unstable and involve many rearrangements and duplications of virus and host sequences [120]. It is generally thought that the E1 and E2 replication proteins are not expressed from integrated genomes but expression of these proteins from co-replicating, extrachromosomal HPV genomes induces recruitment of DDR proteins to the integration loci, resulting in onion skin replication and promoting genetic instability [121,122]. In the absence of the viral replication proteins, genetic instability can be further increased by homologous recombination-related looping mechanisms [120]. Any modification that results in increased expression of the E6 and E7 oncoproteins will further stimulate genetic instability and promote carcinogenesis.

## 4. Conclusions

DDR signaling upon infection is a common strategy utilized by many DNA viruses to recruit factors necessary for replication [74], and, as described here, activation of the DDR plays an important role in the HPV life cycle. As a small virus with limited coding capacity and few replication proteins, HPV needs to utilize the host DNA synthesis machinery to amplify itself. However, it must also reprogram and coordinate multiple cellular signaling pathways to establish an environment conducive to viral replication. Manipulation of the DDR provides a means to efficientlymodulate cell cycle checkpoints as well as recruit and usurp replication factors, especially in differentiated cells. The rapid expansion of the genome copy number during the amplification phase of the viral life cycle requires a replication mechanism with high fidelity and there is evidence that homologous recombination repair pathways may provide a means to both initiate replication and resolve DNA intermediates that occur during this phase of rapid DNA synthesis. Ongoing research in many laboratories should provide further mechanistic insight into the pathways and events that result in successful HPV propagation.
